# Waterpipe Smoking and Regulation in the United States: A Comprehensive Review of the Literature

**DOI:** 10.3390/ijerph120606115

**Published:** 2015-05-29

**Authors:** Linda Haddad, Omar El-Shahawy, Roula Ghadban, Tracey E. Barnett, Emily Johnson

**Affiliations:** 1 College of Nursing, University of Florida, Gainesville, FL 32610, USA; 2 Social and Behavioral Health Department, Virginia Commonwealth University, 830 East Main Street, Richmond, VA 23298, USA; E-Mail: elshahwyo@vcu.edu; 3 School of Nursing, Virginia Commonwealth University, 821 West Franklin Street, Richmond, VA 23284, USA; E-Mail: ghadbanr@vcu.edu; 4 College of Public Health and Health Professions, University of Florida, Gainesville, FL 32610, USA; E-Mail: tebarnett@phhp.ufl.edu; 5 College of Health and Human Performance, University of Florida, Gainesville, FL 32610, USA; E-Mail: ejohnson719@hhp.ufl.edu

**Keywords:** waterpipe, hookah, regulation, policy

## Abstract

*Background*: Researchers in tobacco control are concerned about the increasing prevalence of waterpipe smoking in the United States, which may pose similar risks as cigarette smoking. This review explores the prevalence of waterpipe smoking in the United States as well as the shortcomings of current U.S. policy for waterpipe control and regulation. *Methods*: Researchers conducted a literature review for waterpipe articles dated between 2004 and 2015 using five online databases: MEDLINE, CINHAHL, ScienceDirect, PMC, and Cochrane Library. *Results*: To date, few studies have explored the marketing and regulation of waterpipe smoking in the U.S., which has increased in the last ten years, especially among women, adolescents, and young adults. Data indicate that the majority of waterpipe smokers are unaware of the potential risks of use. In addition, current tobacco control policies do not address waterpipe smoking, enabling tobacco companies to readily market and sell waterpipe products to young adults, who are at risk for becoming lifelong smokers. *Conclusion*: Policy makers in the area of public health need to update existing tobacco regulations to include waterpipe smoking. Similarly, public health researchers should develop public health campaigns and interventions to address the increasing rates of waterpipe smoking in the United States.

## 1. Introduction

The smoke inhaled through water pipes use (WPU) contains toxicants similar to cigarettes such as hydrocarbons, carbon monoxide, and carcinogenic polycyclic aromatic volatile aldehydes [1,2,3]. Despite the fact that the average frequency of WPU in the United States is lower than that of cigarettes, a single WPU session typically lasts for 45 minutes and may produce 50 to 100 times the smoke volume inhaled from a single cigarette [4,5,6,7,8]. Indeed, one study found that once-a-day WP smokers had levels of plasma nicotine concentration comparable to smokers who used 10 cigarettes per day [5,9]. More recent evidence indicates that WPU may have the same health risks as cigarette smoking, such as nicotine addiction, exposure to second hand smoke, and an increased risk for a variety of chronic diseases [10]. Therefore, tobacco control researchers are concerned that WPU has reached a staggering high prevalence rate among young adults [1,11,12]. This prevalence may be even higher among college students and young women [13,14] who perceive WPU as more socially acceptable than cigarette smoking [15,16,17]. Additionally, WPU could provide a gateway to other forms of smoking, which may undermine the advances in tobacco reduction over the last 30 years [18]. In order to potentially reverse this trend, policy makers need reliable scientific information to develop regulations for the marketing, packaging, and consumption of WP in the United States. Thus, this review aims to examine evidence-based research about WPU to inform policy makers and the Food and Drug Administration (FDA) about the needed WPU regulatory actions in the United States.

## 2. Methods

We conducted our literature review between November 2014 and March 2015 via searching for articles published in English between 2005 and 2015, and used the following electronic databases: MEDLINE, CINHAHL, ScienceDirect, PMC, and Cochrane Library. Our search terms were “waterpipe” and its alternative spellings, which were “hookah”, “shisha”, “narghile”, “hubble bubble”, and “goza”. We limited our search to published research studies; however, gray literature, including published abstracts, conference proceeding, theses, dissertations, and government and organization reports, were also identified.

### Study Design

Our search sample was limited to studies conducted in the United States to address the main outcome of our review, and we included all possible study designs except for review articles.

## 3. Results

**Figure 1:**
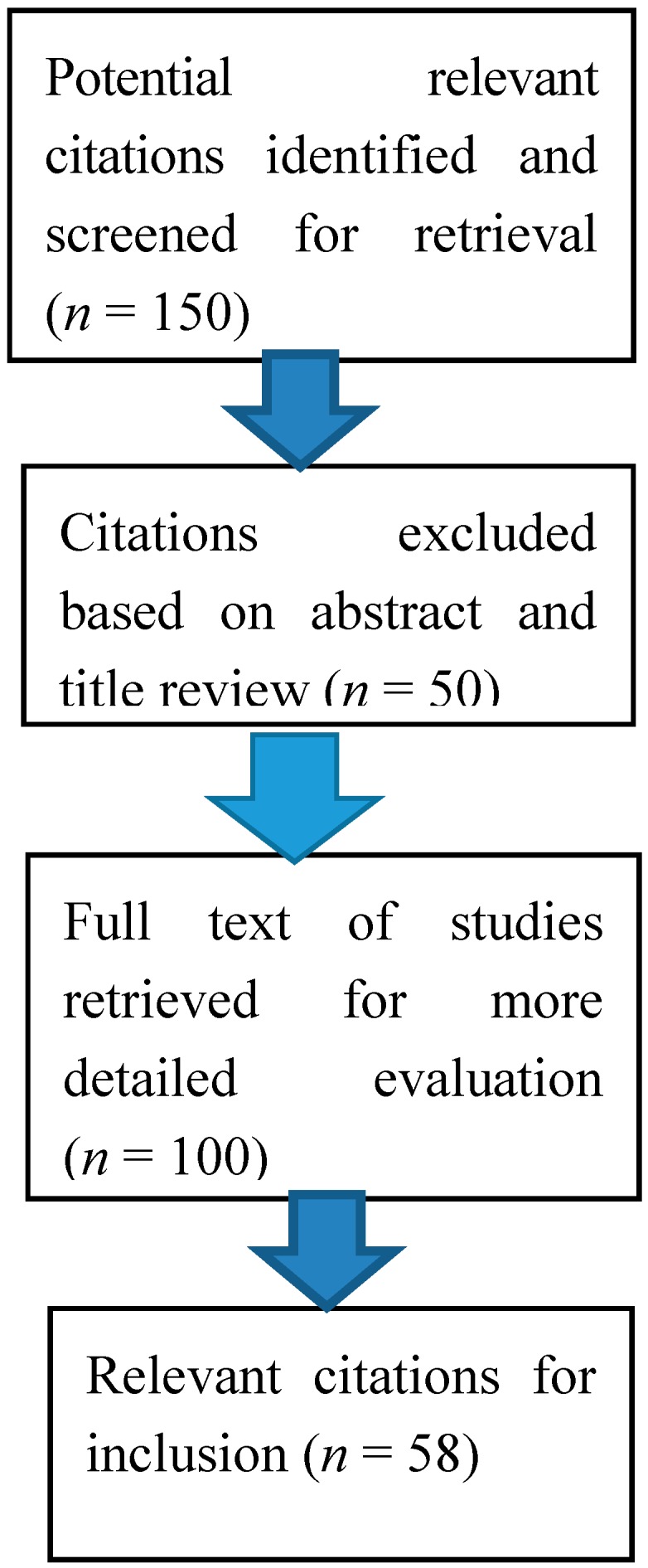
Results of the literature search.

Our searches identified 150 potential relevant papers, of which 100 met inclusion criteria (see Figure 1). Results were comprised of longitudinal and prospective (four), observational/descriptive (cross-sectional, survey) (36), reviews of literature/policies (10), measurement scale (one), and social media studies (seven).

### 3.1. Waterpipe Smoking Trends in the U.S.

Among U.S. high school students, the Center for Disease Control (CDC) reported that cigarette use has dropped by 33%, while use of non-cigarette combustible tobacco products, including WPU, has increased by 123% (2012). Many studies on the prevalence of WPU in the U.S. focused on high school and college students and results from the 2010 Monitoring the Future study reported that among high school seniors, about one in five (17%) males and one in six (15%) females had used waterpipe in the past year [13,14,19,20,21,22,23,24,25,26]. Data indicates that the prevalence of WPU among high school students increased from 11% in 2007 to over 18% [13]. Furthermore, Amrock *et al*., surveyed over 18,000 US adolescents, representing more than 27 million adolescents nationwide, and found that roughly two million adolescents report having ever used waterpipe and 720,000 reported current use [27]. Their results also showed that males were more likely than females to report ever-using waterpipe (8.1% *vs.* 6.6%, respectively), but were not more likely to report using waterpipe in the past 30 days (3.0% *vs.* 2.3%), respectively [27]. However, rates of past use among high school students vary by race, with Whites the most likely (8.4%) and Blacks the least likely (2.3%) to smoke WP [27]. Students of other or multiple races (3.9%) and Hispanics (3.3%) were most likely to report using waterpipe in the past 30 days [27].

One study reported that among all age groups, young adults aged 18–24 have the highest prevalence of WPU (28.6%) in the United States [28]. Moreover, using the National Adult Tobacco Survey, this study also reported that when compared to people aged between 35 and 44 years, young adults in this age group were eight times more likely to have used waterpipe and were 18 times more likely to report being current users [28]. Among all age groups, overall prevalence rates were lowest among non-Hispanic Blacks (3.6%) and highest among non-Hispanic Others or those of mixed races (17.6%) [28]. Furthermore, Salloum *et al*., reported that those with some college education (but with no degree) and those with annual household incomes over $100,000 had the highest rate of past use (12.4% and 12.0%, respectively [28].

Additionally, Arab American teens reported high WPU (12%–15%) [26,29,30] whereas prevalence of WPU among university students ranged widely from 10% to 46% [14,21,31,32,33]. Researchers in the area of tobacco control expect that WPU is likely to increase in young adults because college students view WPU as a socially acceptable group activity. Finally, only one study has examined trends of WPU in U.S. college students with a longitudinal design [22].

Overall, studies in the U.S. indicated that WPU is associated with being male, having peers who smoke waterpipe, and social acceptability [15,34]. Other studies in the U.S., which examined the initiation and pattern of WPU at a single time point [15,23,35,36,37], revealed that WPU had higher social approval than cigarette use had. The gap between males and females is smaller for WPU compared to cigarette smoking. Data indicates that some women prefer WPU to cigarettes, perhaps due to the social environment appeal of WPU or the influence of social media such as Pinterest, which portray WPU in a positive light [38,39]; this could fuel a gender disparity in WPU among women in the US in the near future.

### 3.2. Misperceptions of Harm

Favorable attitudes toward WPU are possibly related to its reported sweet smell and pleasant taste by users; as a result, users view it as an appealing way to spend leisure time socializing with friends [35]. One study utilized the Theory of Reasoned Action to evaluate users’ beliefs regarding WPU, results indicated that favorable intentions toward WPU were a significant predictor of use three months later. These intentions explained 50% of the variance in WPU for study participants, which signifies the importance of those favorable attitudes and “potentially *normalized*” social norms surrounding waterpipe in the initiation of WPU [15,35].

In addition, many Americans are misinformed about the health risks of WPU [21,35,40,41]. WPU is widely perceived to be less harmful and less addictive than cigarette smoking [15,23,35,36,37] due to the erroneous belief that water filters the carcinogens in tobacco smoke [42,43]. In fact, waterpipe smoke contains similar toxins to those found in cigarette smoke, including carbon monoxide, and tar; 82 other toxins have been identified. Thus, WPU may pose similar health risks as cigarette smoking, such as cancer, heart disease, and nicotine addiction, which can affect non-smokers through exposure to secondhand smoke [43,44,45,46,47,48,49,50,51,52]. Two hours of second hand smoke (SHS) exposure in a waterpipe café is considered equivalent to smoking 10 cigarettes per day [39] and exposes users to higher levels of carbon monoxide due to the use of charcoal [53]. Other studies have shown associations between WPU and coronary artery disease [54], lung cancer, low birth weight, and periodontal disease, as well as milder associations between bladder, esophageal cancer, and infertility [55]. Unlike cigarettes, WPU has also been linked to many communicable infectious diseases, including herpes simplex virus and respiratory viruses, which could spread when waterpipe users share hoses.

In response to these misperceptions about health risks, tobacco control researchers examined the perceived health risks of WPU, particularly among young adults [20,35,38,41]. Results of each study were similar: participants perceived WPU to be less harmful than cigarette smoking. This may be because state regulatory agencies have yet to ban waterpipe cafes, which contribute to the misperception that WPU is not as harmful as cigarette smoking. This message may be reinforced further when businesses prominently display, as they are required to do, their favorable health inspection score from their local health department [4,56].

### 3.3. Limited Research

To date, few studies have addressed WPU in relation to its SHS exposure and even fewer have included samples from the United States [2,39,57,58]. Similarly, few published studies have examined the impact of WPU on WP dependence [59]. The majority of the previous studies of WPU in the U.S. have been cross-sectional and all of them were limited to students (high school or college). However, one prospective study followed freshmen women on a monthly basis at a private U.S. college for nine consecutive months during their first academic year of college [22]. This study examined risk and protective factors associated with initiation of WPU and reported that among the participants who reported no pre-college WPU, 23% initiated use during their first year. Results also showed that alcohol use was associated with the initiation of WPU and marijuana predicted the frequency of use. To date, most cited waterpipe studies were conducted in the Middle East [7,8,9,11,41,57,60,61,62,63,64] where WPU has strong cultural roots and may be used in different frequencies and patterns than in the U.S.

### 3.4. Waterpipe Smoking Appeal

WPU has a strong social appeal that is making it more popular among users in different countries, including the U.S. For example, in many countries and in parts of the U.S., cigarette smoking among women is considered unacceptable behavior; however, WPU by women does not carry a similar stigma. International studies have validated this double-standard between cigarette and WPU by women [65,66]. Perhaps due to the lack of stigma, women in several studies reported that WPU has more positive attributes (e.g., social, attractive, traditional, and familiar) than cigarette smoking and thus prefer this form of tobacco use. Considering the results of these studies, it is not surprising that unlike cigarette smoking/addiction, there is no male gender predominance in WPU [67,68,69].

### 3.5. Waterpipe Popularity

Waterpipe cafés are gaining in popularity in the U.S. and internationally [70] for several reasons. First, waterpipe cafés or bars, unlike traditional bars, do not have strict admissions policies on individuals under age 21 because alcohol is not typically served on the premises [56]. This feature attracts many young adults who seek a social activity with friends but are not old enough to enter a traditional bar. Second, WP cafés provide the same social atmosphere as a bar: the communal waterpipe is the conduit for social interaction. In addition, WPU is generally viewed as “cool”, [12,71,72] resulting in peer pressure that may increase the number of individuals who try WPU or who willingly expose themselves to the secondhand smoke in a café setting. In fact, one British study found that WPU increased with time spent in college, whereas the highest level of smoking cigarettes occurred during the first year of school and then decreased. The same study also found that 83.9% of college students were introduced to WPU by a friend, which is likely to occur more often in the U.S. as the number of waterpipe cafés increases.

### 3.6. Waterpipe Social Context

WPU in a café has typical appeal or *social context*, which is defined as the immediate, situational, temporal, and motivational factors that influence behavior, is a key influence in the WPU experience [73]. However, at present, little is known about the social context that influences WPU or waterpipe SHS exposurein the U.S. An understanding of the social context of these settings could help explain when, where, why, and with whom young adults prefer to socialize where WPU is known to occur. It is likely that young adults prefer waterpipe cafes because of the informal, social atmosphere, and that WPU may provide an inexpensive way to spend leisure time with friends.

### 3.7. Online Media Sources of Waterpipe Information

Popular online media platforms, such as Twitter and Facebook, play an increasing role in the communication of public health information [6,74]. A recent study [75] found that Americans often rely on online contacts for health information and assign high credibility to their peers’ assessments and information. Results also indicated that one third (34%) of internet users had read commentaries about health issues in news groups, websites, or blogs. Similarly, one fourth (24%) had read reviews on drugs and treatments and nearly one-fifth (18%) had gone online to find people with similar health concerns in order to seek advice and information [75]. This new pattern of information-seeking is important because public health resources, both accurate and inaccurate, can profoundly influence public understanding, attitudes, and behavior [76].

Indeed, in the virtual world, some sources may appear falsely credible [77] and reliable sources may compete with commercial messages to preclude the delivery of accurate information. Myslin *et al*., examined the content analysis of tobacco-related posts on the popular social media site Twitter to determine sentiment (positive or negative) towards tobacco, including WPU [78]. Researchers analyzed 7362 tobacco-related Twitter posts (*i.e*., ‘tweets’) and noted that keywords such as *hookah* and *shisha* were classified as showing positive sentiment, compared to the negative sentiment associated with keywords such as *nicotine* and *tobacco*. Analogous to other research, these results show that WPU is viewed more favorably than using traditional tobacco products. The researchers also noted that the tweets were not posted by recognized health organizations, indicating that much information on Twitter hails from unverified sources.

In a similar study, Salloum *et al*. [79], utilized data obtained from *Google Trends* (a publically-accessible database that tracks internet search terms) to assess the popularity of waterpipe internet search queries in four English-speaking countries, including the United States. They report that the online popularity of waterpipe searches was highest in the U.S. compared to the other three countries, and that in the U.S. alone, waterpipe shopping searches (searches conducted under the *Google Shopping* Category) increased by 291% between January 2004 and December 2013. They also report that the most common WPU search terms included hookah (approximately 190,000 weekly searches) and shisha (approximately 127,000 weekly searches). The researchers conclude that web-based search queries for waterpipe have steadily increased over the past decade, pointing towards a growing interest in WPU. Thus, researchers in the area of tobacco control must now consider social media in addition to traditional media (e.g., TV, newspaper, web) and offline interpersonal communications to fully understand the conveyance of WP information [77,80,81,82,83]. Ultimately, this understanding will enable researchers to develop public health campaigns about the dangers of WPU, resulting in reduced rates of WPU in the U.S. over time.

### 3.8. Waterpipe Marketing

Despite the growth in popularity of WPU, there is minimal literature about the marketing strategies used to promote waterpipe, or “hookah”, establishments. For example, some venues offer a variety of Mediterranean and American food and even alcohol, whereas others may attract customers through belly dancers, poker nights, musical performances, or free Wi-Fi access. One study found that advertisements for waterpipe cafes on the Internet used text, images, or audio stimuli to promote waterpipe smoking [56]. Notably, these websites advertised that waterpipe smoking was a safe, fun, relaxing, and “tasty” (*i.e*., sweet) way to socialize with friends [56]. None of the cafes or websites required age verification, which may entice minors to waterpipe cafes to consume waterpipe products. In addition, many waterpipe companies target youth by offering multiple flavors in their product lineup, which encourages WPU in this population.

### 3.9. Industry Regulation

Because of increasing WPU among young American adults and poor regulation of the waterpipe industry, researchers are concerned that WPU may lead to the use of other tobacco products over time [80,84,85,86]. Indeed, the growth in WPU indicates a need for waterpipe -specific regulatory policies in the U.S. as well as a need to update existing tobacco laws to include WPU [18,87].

Policy makers in the area of public health should consider the unique aspects of WPU when developing industry regulations. For example, because waterpipe smokers in a café setting do not interact with the packaging, they often do not see the conspicuous warning labels on waterpipe products. Thus, in order to be effective, health-warning labels for waterpipe products would need to be acknowledged by the consumer at point-of-sale, or upon entering the waterpipe café. Also, these cafes, which are considered ‘tobacco retail shops’ in the U.S., are exempt from the smoke-free laws common in large cities [87]. This exemption is problematic for two reasons: first, it may lead to a high level of waterpipe SHS exposureamong the non-smoking customers who attend the cafés [8,41,88,89,90], and second, it may send a message to the public that waterpipe cafes are “safe” and “normal” [91]. To address this loophole, governments should consider adopting smoke-free laws that broadly define “smoking” as the direct burning or indirect heating of any tobacco or plant product intended for inhalation [92]. With this definition, virtually all smoking products, including WPs, would be included in a regulatory smoking ban.

Policy makers should also consider the exclusion of WPU from laws that govern tobacco products in the United States. For example, the Food and Drug Administration Family Smoking and Prevention Control Act specifies (Section 907, titled ‘Tobacco Product Standards’) a ban on flavored cigarettes, but fails to mention waterpipe tobacco [93]. This exclusion is worrisome because flavor and smell are considered primary motives for the initiation of WPU [68], largely because they mask the harsh taste of tobacco and make the product smoother and more enjoyable. Notably, the Food and Drug Administration can extend a flavor ban to any tobacco product without an act of Congress [93], and state and local governments can also pass laws banning the sale of flavored tobacco, which can greatly limit WP tobacco sales [85]. (See Table 1, which shows that most tobacco product laws do not include WPU).

**Table 1:** Summary of federal tobacco product legislation [93].

Legislation	Year	Description
Federal Cigarette Labeling and Advertising Act (FCLAA)	1965	Required warning labels on cigarette packs-“Caution: Cigarette Smoking May Be Hazardous to Your Health”; however, this law did not apply to tobacco-related advertisements
Public Health Cigarette Smoking Act	1970	Banned cigarettes ads on the radio or television
Comprehensive Smoking Education Act (Public Law 98–474)	1984	Required four rotating health warning labels (all listed as Surgeon General's Warnings) on cigarette packages and advertisements
		Required cigarette industry to provide a confidential list of ingredients added to cigarettes manufactured in or imported into the United States
Public Law 100–202	1987	Banned smoking on domestic airline flights scheduled for two hours or less
Public Law 101–164	1989	Banned smoking on domestic airline flights scheduled for six hours or less
Pro-Children Act	1994	Required all federally funded children's services to become smoke-free. Expanded upon 1993 law that banned smoking in Women, Infants, and Children (WIC) clinics
Family Smoking Prevention and Tobacco Control Act (Tobacco Control Act)	2009	Gave FDA authority to regulate the manufacturing, distribution, and marketing of tobacco products
		Required that smokeless tobacco packages and advertisements have larger and more visible and effective warnings. Smokeless tobacco includes tobacco products such as moist snuff, chewing tobacco, and snus.
		Established and enforced restrictions on tobacco advertising and promotions
		Required tobacco companies to disclose what is in their products
		Only included cigarettes, cigarette tobacco, roll-your-own tobacco, and smokeless tobacco
Center for Tobacco Products ban on flavored tobacco	2009	Banned the sale or distribution of any cigarettes containing an artificial or natural flavor other than tobacco. This ban did not apply to menthol.
Regulations Restricting the Sale and Distribution of Cigarettes and Smokeless Tobacco to Protect Children and Adolescents (Under the Tobacco Control Act)	2010	Designed to curb access to cigarettes and smokeless tobacco products to children and adolescents in the United States
		Prohibited the sale of cigarettes, cigarette tobacco, and smokeless tobacco to people younger than 18.
		Prohibited the sale of cigarette packages with fewer than 20 cigarettes.
		Prohibited the sale of cigarettes and smokeless tobacco in vending machines, self-service displays, or other impersonal modes of sales, except in very limited situations.
		Prohibited free samples of cigarettes and limit distribution of smokeless tobacco products
Tobacco Products Deemed To Be Subject to the Food, Drug & Cosmetic Act	In progress	Proposed newly ‘deemed’ products would include electronic cigarettes, cigars, pipe tobacco, certain dissolvables that are not smokeless ‘tobacco’, gels, and waterpipe tobacco

Finally, policy makers could consider reducing access to waterpipe products through an increase in taxes and strict legislation. For example, the current price of waterpipw tobacco is approximately $22 per pound less than the price of cigarette tobacco [85,94]. If policy makers increased the price of waterpipe tobacco to be equal to that of cigarette tobacco, then waterpipe products would be less affordable, particularly to young adults who are most likely to consume them. Policy makers could also reduce access to waterpipe products by regulating their portrayal on social media (e.g., Twitter and Facebook) [95,96] and retail webpages, as well as limiting access to online vendors, and creating age restrictions for the sale of waterpipe products. All of these regulatory methods show great promise in reducing the rates of WPU over time, particularly among heavy consumers of waterpipe products, such as women and young adults.

## 4. Conclusions

WPU is increasing in popularity due to few regulatory laws and public attraction to this new and “cool” form of smoking. Presently, there is ample international research on WPU, but few studies have been conducted in the U.S. about the marketing and regulation of waterpipe as well as current trends in use, we provide a list of included studies and their design in the appendix (Table A1). Thus, more research is needed that targets vulnerable populations (e.g., young adults and/or women in college towns) to determine their (a) current perceptions of the health risks of WPU; (b) access to and quality of online media information about WPU; and (c) willingness to engage in a public health intervention to reduce WPU. The same successful strategies that have been used for cigarette smoking could be applied to WPU. Our findings show there is much room for development of waterpipe policy and until the FDA extends its regulatory authority to the waterpipe industry, state policy makers need to revise existing cigarette-specific legislation to include WPU. Such changes may profoundly affect rates of WPU over time.

## Appendix

**Table A1:** List of included studies and their design.

Authors	Design
Shishani, K.; Howell, D.; McPherson, S.; Roll, J. Young adult waterpipe smokers: Smoking behaviors and associated subjective and physiological effects. *Addict. Behav.* **2014**, *39*, 1113–1119.	Longitudinal and Prospective
Villanti, A.C.; Cobb, C.O.; Cohen, A.M.; Williams, V.F.; Rath, J.M. Correlates of hookah use and predictors of hookah trial in U.S. young adults. *Am. J. Prev. Med.* **2015**.	
Fielder, R.L.; Carey, K.B.; Carey, M.P. Predictors of initiation of hookah tobacco smoking: A one-year prospective study of first-year college women. *Psychol. Addict. Behav.* **2012**, *26*, 963–968.	
Fielder, R.L.; Carey, K.B.; Carey, M.P. Hookah, cigarette, and marijuana use: A prospective study of smoking behaviors among first-year college women. *Addict. Behav.* **2013**, *38*, 2729–2735.	

Rezk-Hanna, M.; Macabasco-O’Connell, A.; Woo, M. Hookah smoking among young adults in Southern California. *Nurs. Res.* **2014**, *63*, 300–306.	Observational/Descriptive (cross-sectional, survey)
Amrock, S.M.; Gordon, T.; Zelikoff, J.T.; Weitzman, M. Hookah use among adolescents in the United States: Results of a national survey. *Nicotine Tob. Res.* **2014**, *16*, 231–237.	
Linde, B.D.; Ebbert, J.O.; Pasker, C.K.; Talcott, G.W.; Schroeder, D.R.; Hanson, A.C.; Klesges, R.C. Prevalence and predictors of hookah use in US air force military recruits. *Addict. Behav.* **2015**, *47*, 5–10.	
Palamar, J.J.; Zhou, S.; Sherman, S.; Weitzman, M. Hookah use among US high school seniors. *Pediatrics* **2014**, *134*, 227–234.	
Goodwin, R.D.; Grinberg, A.; Shapiro, J.; Keith, D.; McNeil, M.P.; Taha, F.; Jiang, B.; Hart, C.L. Hookah use among college students: Prevalence, drug use, and mental health. *Drug Alcohol Depen.* **2014**, *141*, 16–20.	
Grifith, M.A.; Ford, E.W. Hookah Smoking: Behaviors and beliefs among young consumers in the United Staes. *Soc. Work Pub. Health* **2014**, *29*, 17–26.	
Primack, B.A.; Longacre, M.R.; Beach, M.L.; Adachi-Mejia, A.M.; Titus, L.J.; Dalton, M.A. Association of established smoking among adolescents with timing of exposure to smoking depicted in movies. *J. Natl. Cancer Inst.* **2012**, *104*, 549–555.	

Rice, V.H.; Weglicki, L.S.; Templin, T.; Jamil, H.; Hammad, A. Intervention effects on tobacco use in Arab and non-Arab American adolescents. *Addict. Behav.* **2010**, *35*, 46–48.	
Primack, B.; Sidani, J.; Shadel, W.; Eissenberg, T. Prevalence of and associations with waterpipe tobacco smoking among U.S. University students. *Ann. Behav. Med.* **2008**, *36*, 81–86.	
Sterling, K.L.; Mermelstein, R. Examining hookah smoking among a cohort of adolescent ever smokers. *Nicotine Tob. Res.* **2011**, *13*, 1202–1209.	
Lipkus, I.M.; Eissenberg, T.; Schwartz-Bloom, R.D.; Prokhorov, A.V.; Levy, J. Affecting perceptions of harm and addiction among college waterpipe tobacco smokers. *Nicotine Tob. Res.* **2011**, *13*, 599–610.	
Maziak, W.; Ward, K.D.; Eissenberg, T. Factors related to frequency of narghile (waterpipe) use: The first insights on tobacco dependence in narghile users. *Drug Alcohol Depend.* **2004**, *76*, 101–106.	
Sukaina Alzyoud, L.H.; Omar, E.S.; Roula, G.; Khalid, K.; Khalid, A.A.; Yan, J. Patterns of waterpipe use among Arab immigrants in the USA: A pilot study *Br. J. Med. Med. Res.* **2014**, *4*, 11.	
Primack, B.A.; Shensa, A.; Kim, K.H.; Carroll, M.V.; Hoban, M.T.; Leino, E.V.; Eissenberg, T.; Dachille, K.H.; Fine, M.J. Waterpipe smoking among U.S. University students. *Nicotine Tob. Res.* **2013**, *15*, 29–35.	
Barnett, T.E.; Forrest, J.R.; Porter, L.; Curbow, B.A. A multiyear assessment of hookah use prevalence among Florida high school students. *Nicotine Tob. Res.* **2014**, *16*, 373–377.	
Barnett, T.; Smith, T.; He, Y.; Soule, E.; Curbow, B.; Tomar, S.; McCarty, C. Evidence of emerging hookah use among university students: A cross-sectional comparison between hookah and cigarette use. *BMC Public Health* **2013**, *13*, 302.	
Noonan, D.; Patrick, M.E. Factors associated with perceptions of hookah addictiveness and harmfulness among young adults. *Subst. Abuse* **2013**, *34*, 83–85.	
Sidani, J.E.; Shensa, A.; Primack, B.A. Substance and hookah use and living arrangement among fraternity and sorority members at US colleges and universities. *J. Commun. Health* **2013**, *38*, 238–245.	
Abughosh, S.; Wu, I.H.; Peters, J.R. Predictors of persistent waterpipe smoking among university students in the United States. *Epidemiology: Open Access* **2011**, *01*.	
Noonan, D.; Kulbok, P.; Yan, G. Intention to smoke tobacco using a waterpipe among students in a southeastern U.S. College. *Public Health Nurs.* **2011**, *28*, 494–502.	
Primack, B.A.; Kim, K.H.; Shensa, A.; Sidani, J.E.; Barnett, T.E.; Switzer, G.E. Tobacco, marijuana, and alcohol use in university students: A cluster analysis. *J. Am. Coll. Health* **2012**, *60*, 374–386.	
Rice, V.H.; Weglicki, L.S.; Templin, T.; Hammad, A.; Jamil, H.; Kulwicki, A. Predictors of Arab American adolescent tobacco use. *Merrill Palmer Q (Wayne State Univ Press)* **2006**, *52*, 327–342.	
Jamil, H.; Templin, T.; Fakhouri, M.; Rice, V.H.; Khouri, R.; Fakhouri, H. Comparison of personal characteristics, tobacco use, and health states in Chaldean, Arab American, and non-Middle Eastern white adults. *J. Immigr. Minor. Health* **2009**, *11*, 310–317.	
Primack, B.A.; Fertman, C.I.; Rice, K.R.; Adachi-Mejia, A.M.; Fine, M.J. Waterpipe and cigarette smoking among college athletes in the United States. *J. Adolescent Health* **2010**, *46*, 45–51.	
Backinger, C.L.; Fagan, P.; O’Connell, M.E.; Grana, R.; Lawrence, D.; Bishop, J.A.; Gibson, J.T. Use of other tobacco products among U.S. adult cigarette smokers: Prevalence, trends and correlates. *Addict. Behav.* **2008**, *33*, 472–489.	
Noonan, D.; Kulbok, P.A. Beliefs and norms associated with smoking tobacco using a waterpipe among college students. *J. Addict. Nurs.* **2012**, *23*, 123–128.	
Nuzzo, E.; Shensa, A.; Kim, K.H.; Fine, M.J.; Barnett, T.E.; Cook, R.; Primack, B.A. Associations between hookah tobacco smoking knowledge and hookah smoking behavior among US college students. *Health Edu. Res.* **2013**, *28*, 92–100.	
Primack, B.A.; Walsh, M.; Bryce, C.; Eissenberg, T. Water-pipe tobacco smoking among middle and high school students in Arizona. *Pediatrics* **2009**, *123*, e282–e288.	
Jacob, P., 3rd; Abu Raddaha, A.H.; Dempsey, D.; Havel, C.; Peng, M.; Yu, L.; Benowitz, N.L. Nicotine, carbon monoxide, and carcinogen exposure after a single use of a water pipe. *Cancer Epidemiol. Biomarkers Prev.* **2011**, *20*, 2345–2353.	
Shihadeh, A.L.; Eissenberg, T.E. Significance of smoking machine toxicant yields to blood-level exposure in water pipe tobacco smokers. *Cancer Epidemiol. Biomarkers Prev.* **2011**, *20*, 2457–2460.	
Islami, F.; Nasseri-Moghaddam, S.; Pourshams, A.; Poustchi, H.; Semnani, S.; Kamangar, F.; Etemadi, A.; Merat, S.; Khoshnia, M.; Dawsey, S.M., *et al.* Determinants of gastroesophageal reflux disease, including hookah smoking and opium use- a cross-sectional analysis of 50,000 individuals. *PloS One* **2014**, *9*, e89256.	
Barnett, T.E.; Curbow, B.A.; Soule, E.K., Jr.; Tomar, S.L.; Thombs, D.L. Carbon monoxide levels among patrons of hookah cafes. *Am. J. Prev. Med.* **2011**, *40*, 324–328.	
Akl, E.A.; Jawad, M.; Lam, W.Y.; Co, C.N.; Obeid, R.; Irani, J. Motives, beliefs and attitudes towards waterpipe tobacco smoking: A systematic review. *Harm. Reduct. J.* **2013**, *10*, 12.	
Sharma, E.; Beck, K.H.; Clark, P.I. Social context of smoking hookah among college students: Scale development and validation. *J. Am. College Health* **2013**, *61*, 204–211.	
Myslin, M.; Zhu, S.H.; Chapman, W.; Conway, M. Using Twitter to examine smoking behavior and perceptions of emerging tobacco products. *J. Med. Intern. Res.* **2013**, *15*, e174.	
Salloum, R.G.; Thrasher, J.F.; Kates, F.R.; Maziak, W. Water pipe tobacco smoking in the United States: Findings from the National Adult Tobacco Survey. *Prev. Med.* **2015**, *71*, 88–93.	
Salloum, R.G.; Thrasher, J.F.; Kates, F.R.; Maziak, W. Waterpipe tobacco smoking in the United States: Findings from the National Adult Tobacco Survey. *Prev. Med.* **2015**, *71*, 88–93.	Review of literature/policies
Primack, B.A.; Hopkins, M.; Hallett, C.; Carroll, M.V.; Zeller, M.; Dachille, K.; Kim, K.H.; Fine, M.J.; Donohue, J.M.U.S. Health policy related to hookah tobacco smoking. *Am. J. Public Health* **2012**, *102*, e47–e51.	
Akl, E.A.; Gunukula, S.K.; Aleem, S.; Obeid, R.; Jaoude, P.A.; Honeine, R.; Irani, J. The prevalence of waterpipe tobacco smoking among the general and specific populations: A systematic review. *BMC Public Health* **2011**, *11*, 244.	
WHO Study Group on Tobacco Product Regulation. *Advisory note: Waterpipe Tobacco Smoking: Health Effects, Research Needs and Recommended actions by Regulators*; World Health Organization: Geneva, Switzerland, 2005.	
Chaouachi, K. Hookah (shisha, narghile) smoking and environmental tobacco smoke: A critical review of the relevant literature and the public health consequences. *Int. J. Environ. Res. Public Health* **2009**, *6*, 798–843.	
Griffiths, M.A.; Harmon, T.R.; Gilly, M.C. Hubble bubble trouble: The need for education about and regulation of hookah smoking. *J Pub. Pol. Mark.* **2011**, *30*, 119–132.	
Morris, D.S.; Fiala, S.C.; Pawlak, R. Opportunities for policy interventions to reduce youth hookah smoking in the United States. *Prev. Chronic Dis.* **2012**, *9*, E165.	
McCarthy, M. FDA moves to regulate e-cigarettes and pipe and hookah tobacco. *BMJ* **2014**, *348*, g2952.	
Maher, J.E.; Morris, D.S.; Girard, K.E.; Pizacani, B.A. Consequences of clean indoor air exemptions in Oregon: The hookah story. *Tob. Control* **2014**, *23*, 195–196.	
Jawad, M.; McEwen, A.; McNeill, A.; Shahab, L. To what extent should waterpipe tobacco smoking become a public health priority? *Addiction* **2013**, *108*, 1873–1884.	
Elias, W.; Assy, N.; Elias, I.; Toledo, T.; Yassin, M.; Bowirrat, A. The detrimental danger of water-pipe (hookah) transcends the hazardous consequences of general health to the driving behavior. *J. Transl. Med.* **2012**, *10*, 126.	
Sharma, E.; Beck, K.H.; Clark, P.I. Social context of smoking hookah among college students: scale development and validation. *J. Am. College Health* **2013**, *61*, 204–211.	Measurement scale
Kassem, N.O.; Daffa, R.M.; Liles, S.; Jackson, S.R.; Kassem, N.O.; Younis, M.A.; Mehta, S.; Chen, M.; Jacob, P., 3rd; Carmella, S.G.; *et al.* Children’s exposure to secondhand and thirdhand smoke carcinogens and toxicants in homes of hookah smokers. *Nicotine Tob. Res.* **2014**, *16*, 961–975.	Health effect
Primack, B.A.; Rice, K.R.; Shensa, A.; Carroll, M.V.; DePenna, E.J.; Nakkash, R.; Barnett, T.E. U.S. Hookah tobacco smoking establishments advertised on the internet. *Am. J. Prev. Med.* **2012**, *42*, 150–156.	Social media studies
Salloum, R.G.; Osman, A.; Maziak, W.; Thrasher, J.F. How popular is waterpipe tobacco smoking? Findings from internet search queries. *Tob. Control* **2014**.	
Carroll, M.V.; Shensa, A.; Primack, B.A. A comparison of cigarette- and hookah-related videos on YouTube. *Tob. Control* **2013**, *22*, 319–323.	
Brockman, L.N.; Pumper, M.A.; Christakis, D.A.; Moreno, M.A. Hookah’s new popularity among US college students: A pilot study of the characteristics of hookah smokers and their facebook displays. *BMJ Open* **2012**, *2*, doi:10.1136/bmjopen-2012-001709.	
